# Deep learning guided optimization of human antibody against SARS-CoV-2 variants with broad neutralization

**DOI:** 10.1073/pnas.2122954119

**Published:** 2022-03-01

**Authors:** Sisi Shan, Shitong Luo, Ziqing Yang, Junxian Hong, Yufeng Su, Fan Ding, Lili Fu, Chenyu Li, Peng Chen, Jianzhu Ma, Xuanling Shi, Qi Zhang, Bonnie Berger, Linqi Zhang, Jian Peng

**Affiliations:** ^a^NexVac Research Center, Comprehensive AIDS Research Center, Center for Infectious Disease Research, Department of Basic Medical Sciences, School of Medicine, Tsinghua University, Beijing 100084, China;; ^b^HeliXon Limited, Beijing 100084, China;; ^c^Department of Computer Science, University of Illinois at Urbana–Champaign, Champaign, IL 61822;; ^d^Computer Science & Artificial Intelligence Laboratory, Massachusetts Institute of Technology, Cambridge, MA 02139;; ^e^Department of Mathematics, Massachusetts Institute of Technology, Cambridge, MA 02139;; ^f^Institute for Industry AI Research, Tsinghua University, Beijing 100084, China

**Keywords:** computational biology, deep learning, geometric neural networks, SARS-CoV-2 variants, broadly neutralizing antibodies

## Abstract

The ability of viruses to mutate and evade the human immune system and neutralizing antibodies remains an obstacle to antiviral and vaccine development. Many neutralizing antibodies, including some approved for emergency use authorization (EUA), reduced or lost activity against severe acute respiratory syndrome coronavirus 2 (SARS-CoV-2) variants. Here, we introduce a geometric deep learning algorithm that efficiently enhances antibody affinity to achieve broader and more potent neutralizing activity against such variants. We demonstrate the utility of our approach on a human antibody P36-5D2, which is effective against SARS-CoV-2 Alpha, Beta, and Gamma but not Delta. We show that our geometric neural network model optimizes this antibody’s complementarity-determining region (CDR) sequences to improve its binding affinity against multiple SARS-CoV-2 variants. Through iterative optimization of the CDR regions and experimental measurements, we enable expanded antibody breadth and improved potency by ∼10- to 600-fold against SARS-CoV-2 variants, including Delta. We have also demonstrated that our approach can identify CDR changes that alleviate the impact of two Omicron mutations on the epitope. These results highlight the power of our deep learning approach in antibody optimization and its potential application to engineering other protein molecules. Our optimized antibodies can potentially be developed into antibody drug candidates for current and emerging SARS-CoV-2 variants.

## Significance

SARS-CoV-2 continues to evolve through emerging variants, more frequently observed with higher transmissibility. Despite the wide application of vaccines and antibodies, the selection pressure on the Spike protein may lead to further evolution of variants that include mutations that can evade immune response. To catch up with the virus’s evolution, we introduced a deep learning approach to redesign the complementarity-determining regions (CDRs) to target multiple virus variants and obtained an antibody that broadly neutralizes SARS-CoV-2 variants.

Severe acute respiratory syndrome coronavirus 2 (SARS-CoV-2) has spread worldwide over the past 2 y, causing hundreds of millions of confirmed infections and millions of deaths (1). The receptor-binding domain (RBD) of the SARS-CoV-2 virus spike protein initiates binding to the host receptor, angiotensin converting enzyme 2 (ACE2) (2–6), and serves as an initial essential step in viral–cell membrane fusion, as well as a potential target for neutralizing antibodies (7–10). Neutralizing antibodies that target RBD have already shown therapeutic and clinical value (11–17).

However, reduced sensitivity of SARS-CoV-2 variants to antibody and serum neutralization has been widely observed (18–21). For example, the B.1.617 lineage, also known as the Delta variant, contains two mutations (L452R and T478K) in the RBD that facilitate viral escape—the ability of viruses to evade the immune system and cause disease (22). The L452R mutation is located at the periphery of the receptor binding motif (RBM) and is found to reduce neutralizing activity by antibodies. The T478K mutation in the RBD, located within the epitope region in the RBM, is also associated with antibody escape. There has been striking evidence of antibodies that have been greatly affected, or even have lost their neutralizing activity altogether, by viral escape (23–26).

Experimental methods to improve antibody binding and neutralization have been developed. In vitro affinity maturation methods, such as random mutagenesis with display technologies, has been shown to improve antibody binding against target proteins, but such approaches are time consuming and labor intensive (27–32). Targeted optimization toward one particular variant may also result in loss of neutralizing activity against other variants. Efficient optimization of antibodies that confer broad and potent neutralizing activity against diverse variants is therefore urgently needed.

Here, we develop and apply a deep learning framework to efficiently optimize antibodies to achieve broader and more potent neutralizing activity against SARS-CoV-2 variants. Based on a large collection of antibody–antigen complex structures and binding affinity data, we trained a geometric neural network model, recently developed in computer vision, that effectively extracts interresidue interaction features and makes predictions of changes in binding affinity due to single or multiple amino acid substitutions to the antigen. To search for favorable complementarity-determining region (CDR) mutations that potentially improve antibody binding, we also simulate an in silico ensemble of predicted complex structures with CDR mutations to obtain a robust estimation of the free energy change, also known as ΔΔG. Compared to traditional approaches, the deep learning search space is theoretically much larger and is also easily applicable in targeting multiple variants simultaneously via multiobjective optimization.

To demonstrate the utility of our approach, we sought to optimize a human neutralizing antibody P36-5D2, which was initially isolated from a convalescent patient, and demonstrated reasonably strong potency and breadth against Alpha, Beta, and Gamma (33) but not Delta, due to Delta’s L452R but not T478K mutation through computational structure analysis. We applied our deep learning model to predict CDR sequences that potentially improved binding affinity against the Delta variant while maintaining activity against Alpha, Beta, and Gamma. Through an iterative process of modeling and experimental validation, we were able to obtain six optimized antibodies with substantially improved potency of about 10- to 600-fold against multiple variants, including Delta. We also provide initial promising studies on Omicron. These results highlight the power of deep learning approaches for antibody optimization and their potential application to a wide range of other protein molecules. The optimized antibodies presented here also have the potential to be further developed as antibody drug candidates against SARS-CoV-2 variants.

## Results

### Neural Network Architecture.

We tapped neural networks because they are known as universal approximators for learning complex nonlinear mappings from high-dimensional data with generalizability. Among many successful neural network architectures, attention-based networks are extremely powerful for modeling complicated interactions between entities, making them suitable for modeling proteins whose functions are largely determined by interactions between amino acids (34–37).

**Fig. 1.**
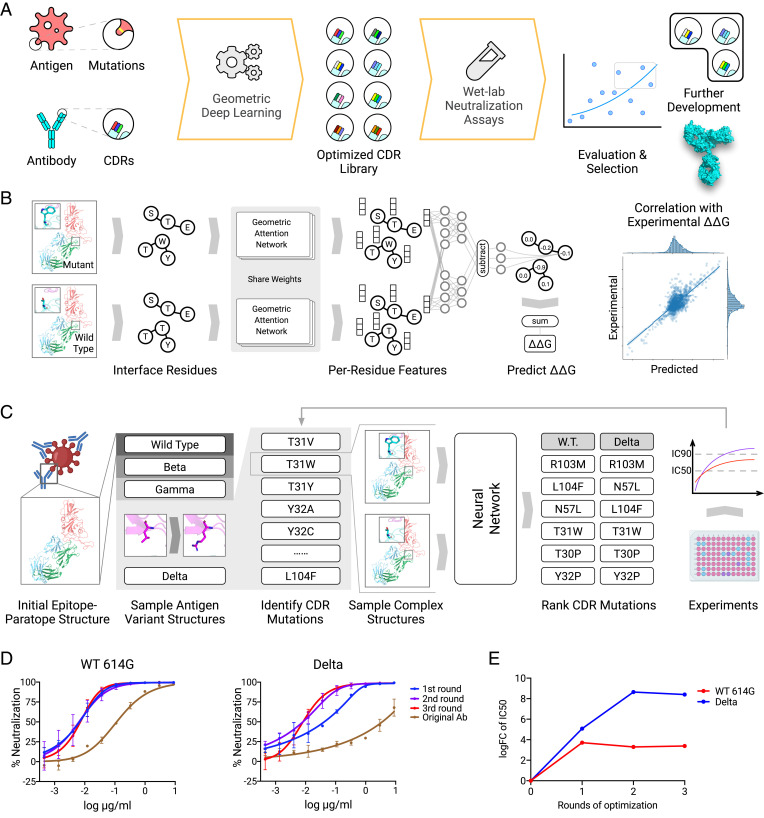
Deep learning guided antibody optimization platform. (*A*) Overview of the pipeline. It demonstrates the computational/experimental feedback loop to refine antibody design. (*B*) Geometric deep learning model. The WT complex and the mutated complex structures are encoded using a shared geometric attention network. The effect of mutation measured by ΔΔG is then predicted by a network that compares features of the two complexes. (*C*) P36-5D2 antibody optimization. Given the complex structure, we first simulate different variants and then evaluate potential CDR mutations that will improve binding by predicted ΔΔG values. Mutants with top ΔΔG scores are examined in laboratory experiments, and those with neutralizing potency are combined for the next round of optimization. (*D*) Optimization improves neutralization ability against SARS-CoV-2 and Delta variant. (*E*) The log fold changes of IC_50_ relative to the original antibody.

We developed an attention-based geometric neural network architecture to learn the mutational effect on protein–protein interactions from three-dimensional protein complex structures (Fig. 1). The geometric part of the model learns a vector embedding for each residue by considering the proximity of its surrounding atoms. Based on these learned geometric embeddings, the attention network learns to identify key residue pairs near the protein interface contributing to binding affinity. Specifically, for each residue in a protein complex, the network first identifies the importance of other residues via the attention mechanism and aggregates information including spatial proximity and physicochemical properties from them. The aggregated information thus can encode the environment as well as interaction features for each residue. To estimate the effect of mutation(s), we first predict the structure of mutated protein complexes by repacking sidechains around mutation sites and encode both wild-type (WT) and mutated complexes using the network to obtain both WT and mutant embeddings. Then, additional neural network layers compare the two embeddings to predict the effect of mutation measured by ΔΔG. This model was evaluated by split-by-complex fivefold cross-validation over the Structural Kinetic and Energetic database of Mutant Protein Interactions (SKEMPI) V2.0 dataset (38). A subset consisting of 1,131 single-point mutations (S1131) (39) was used to benchmark the model and other baselines. The Pearson correlations between the predicted ΔΔG values by different methods and real ΔΔG are reported in *SI Appendix*, Table S1. An additional subset consisting of multipoint mutations (M1707) (40) was also used as a benchmark. We found that our model is able to make predictions with moderate to high correlation with experimental binding data and also outperforms GeoPPI (41), the current state-of-the-art method, as well as a few other recently proposed methods for predicting single-mutation effects. To further evaluate mutated antibody–antigen complexes, we additionally used two other methods, Rosetta and GeoPPI, that also predict mutational effects on stability and binding (41–43), in order to build an ensemble method to evaluate single and higher-order mutations. Our ensemble method is thus able to identify modified antibody sequences that potentially improve antigen binding.

### Antibody of Choice.

We sought to experiment with an antibody that has an epitope with highly conserved regions, which makes it a good candidate to become potent against multiple SARS-CoV-2 variants. We focused on a broad neutralizing antibody P36-5D2, isolated from convalescent patients, known to neutralize Alpha, Beta, and Gamma (33). However, P36-5D2 activity against Delta is reduced by ∼40-fold, relative to that of WT SARS-CoV-2 (Fig. 1*D*). Structural information on the epitope of P36-5D2 indicated that such a reduction was likely attributed to L452R and T478K mutations found in the RBD of Delta. The longer sidechain of the L452R mutation might have exerted steric interference for antibody recognition.

### Generalization to Delta Variant.

To improve the neutralizing activity of P36-5D2 against the Delta variant, we applied our deep learning method to predict and identify optimized antibody mutations. While the initial structure of P36-5D2 bound with the RBD, the neutralizing activity of P36-5D2 against SARS-CoV-2 WT, Beta, Gamma, and Delta was then utilized to analyze and select CDR mutations. Based on those data, our method generated an in silico mutation library of antibody CDRs, ranked by trained geometric neutral networks in such a way that they should not only improve antibody binding to the Delta RBD but also maintain binding to the RBD of other variants of concern (VOC). The predicted top-ranked mutations were selected for experimental verification via pseudotyped virus neutralization assays (*Methods*). A total of four rounds of optimization for P36-5D2 were performed to select the optimized antibodies with best neutralizing potency and breadth. During experimental evaluation, we also included REGN-10987 (44), an ultrapotent antibody approved for emergency use authorization, as the positive control.

#### First-round computational results.

We first sought to narrow down which optimized antibodies to experimentally test, the number determined by our experimental capacity. According to the model’s predictions, 12 top-ranked single mutations were selected and introduced into the original antibody P36-5D2. These mutations included T30P, T31W, and Y32P in HCDR1; N52D, A53F, N55L, and N57L in HCDR2; G100P, R103M, L104F, and Q105F in HCDR3; and S30Y in LCDR1. Out of the 12 single-mutation sites, only 4 (T30, T31, R103, and Q105) were positioned on the paratope of P36-5D2 bound to the RBD, while the remaining 8 were outside and did not directly interact with the RBD. To evaluate the neutralizing breadth and potency of the optimized antibodies, we used an established panel of 10 pseudotyped viruses, including SARS-CoV-2 WT 614G, four VOCs—Alpha, Beta, Gamma, Delta—as well as Delta Plus, two VOIs—Kappa, Epsilon which carried L452R, and Eta which carried E484K—and the variant N439K which effected reference antibody REGN10987.

#### First-round experimental results.

**Fig. 2.**
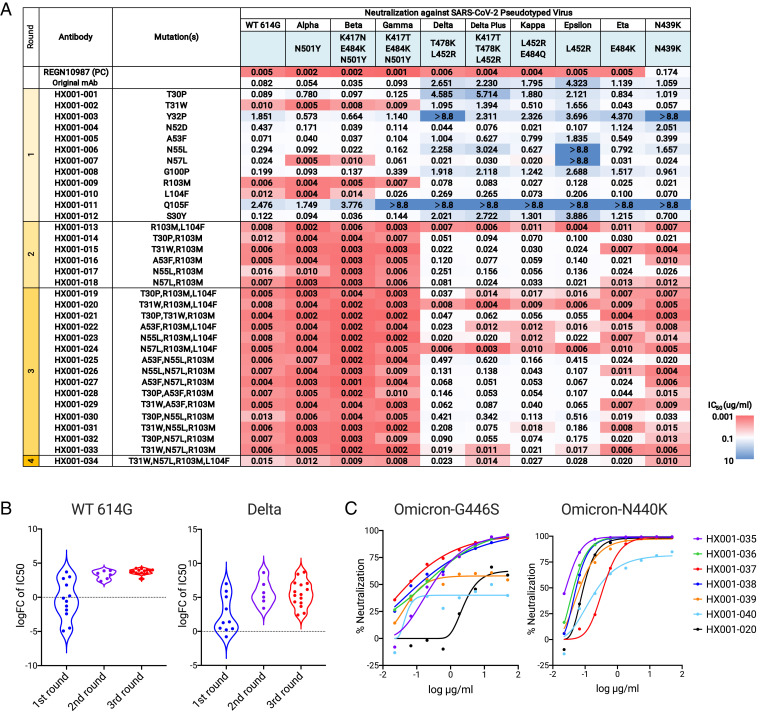
Evaluation of the neutralization level of optimized antibodies. (*A*) IC_50_ values of optimized antibodies against pseudotyped SARS-CoV-2 variants. Mutations of each optimized antibody and mutations of each variant on RBD are indicated. Results were calculated from three independent experiments. (*B*) The log fold changes of IC_50_ relative to the original antibody against SARS-CoV-2 and Delta variants are calculated. (*C*) Neutralization curves of optimized against pseudotyped SARS-CoV-2 carrying Omicron mutations N440K or G446S.

In the first-round neutralization shown in Fig. 2, the original antibody P36-5D2 neutralizes SARS-CoV-2 WT, Alpha, Beta, and Gamma, but is affected by Delta and other viruses carrying L452R, consistent with our structural analysis and predictions. R103M in HCDR3, which was the most surprising mutation, significantly improved neutralizing activity of P36-5D2 against all 10 pseudotyped viruses with average half-maximal inhibitory concentration (IC_50_) reaching 0.038 μg/mL. Four mutations, T31W in HCDR1, A53F, and N57L in HCDR2, and L104F in HCDR3, also improved the overall neutralizing activity, with average IC_50_ of 0.479 μg/mL, 0.547 μg/mL, 0.025 μg/mL, and 0.104 μg/mL, respectively. Three mutations, N52D, N55L in HCDR2, and G100P in HCDR3, obtained enhanced neutralization against Delta but reduced neutralization against WT. The four mutations T30P, Y32P in HCDR1, Q105F in HCDR3, and S30Y in LCDR1 had negligible impact on the neutralizing activity of P36-5D2.

#### Second round: From single to double mutations improves potency.

Based on the first-round results, we combined single mutations to create double mutants and selected them also according to ΔΔG predictions. As R103M was the best single mutation in the first round, the double mutations were designed to pair R103M with another mutation, including L104F, T30P, T31W, A53F, N55L, or N57L in HCDRs. Each of those six double mutations achieved over an order of magnitude improvement in neutralizing activity over the best single mutation R103M in the first round, especially for HX001-013 (R103M and L104F) and HX001-015 (R103M and T31W), for which average IC_50_ values increased to 0.007μg/mL and 0.013μg/mL, respectively (Fig. 2). HX001-013 has an IC_50_ similar to the reference antibody REGN10987 and is more potent than REGN10987 in neutralizing the N439K variant.

#### Third-round optimization identifies potent triple mutations.

Following a similar strategy, single mutations proven to improve potency and breadth in the first and second rounds of optimization were combined to create 15 antibodies with triple mutations. They included R103M and an additional two selected from the following six residues: T30P, T31W, A53F, A55L, N57L, and L104F. As expected, optimized antibodies with three mutations demonstrated improved potent neutralizing activity against the pseudotyped viruses, especially HX001-020 (R103M, T31W, and L104F), HX001-024 (R103M, N57L, and L104F), and HX001-033 (R103M, T31W, and N57L) with average IC_50_ of 0.006 μg/mL, 0.006μg/mL, and 0.010μg/mL, respectively (Fig. 2). HX001-020 and HX001-024 displayed IC_50_ values similar to the reference antibody REGN10987 in general and were better than REGN10987 in neutralizing the N439K mutant.

#### Fourth round investigates quadruple mutants.

Since optimized antibodies with triple mutations such as T31W/N57L/R103M, R103M/T31W/N57L, and R103M/N57L/L104F all demonstrated strong neutralizing activity in round three, we constructed HX001-034, a quadruple mutant, to include all four single mutations. HX001-034 had potent neutralizing activity, with an IC_50_ of 0.017μg/mL, yet was slightly weaker than three mutation combinations: HX001-020, HX001-024, and HX001-033 (Fig. 2).

### Optimized Antibodies Neutralizing Delta.

To experimentally test our predicted mutations, we compared the binding affinity of the original antibody and optimized ones against the SARS-CoV-2 WT RBD monomer and Delta RBD monomer by surface plasmon resonance (SPR) (Table 1). The dissociation constant (K_D_) of the optimized antibodies to the SARS-CoV-2 WT RBD was 1.2 nM to 0.42 nM, which is 20- to 50-fold stronger than the original P36-5D2 antibody. While the off-rate (k_d_) value of the original antibody against WT RBD was around 10^−2^, the off-rate (k_d_) values of the six optimized antibodies all reached 10^−3^, signifying a longer half-life binding period and higher binding stability. Antibodies HX001-020, HX001-024, HX001-033, and HX001-034 with three or four mutations were also stronger than HX001-013 with only two mutations. The increase in binding affinity may contribute to the increased neutralizing activity of these antibodies against SARS-CoV-2 WT and variants. Notably, the dissociation constant of optimized antibodies with the SARS-CoV-2 Delta RBD was 6.22 nM to 26.4 nM, which is twofold stronger than or similar to P36-5D2, while the K_D_ of optimized antibodies with the SARS-CoV-2 Delta RBD is still slightly weaker than with WT RBD.

**Table 1.** Binding kinetics of original Ab and optimized antibodies with SARS-CoV-2 RBD measured by SPR

mAb name	WT RBD			Delta RBD		
	ka (1/Ms)	kd (1/s)	KD (nM)	ka (1/Ms)	kd (1/s)	KD (nM)
Original antibody	1.37 × 10^6^	3.14 × 10^−2^	22.9	2.58 × 10^6^	2.94 × 10^−2^	11.4
HX001-013	1.49 × 10^6^	1.79 × 10^−3^	1.20	2.42 × 10^6^	1.89 × 10^−2^	7.81
HX001-015	3.94 × 10^6^	2.53 × 10^−3^	0.64	1.50 × 10^6^	3.97 × 10^−2^	26.4
HX001-020	4.50 × 10^6^	2.51 × 10^−3^	0.56	2.73 × 10^6^	2.03 × 10^−2^	7.44
HX001-024	2.77 × 10^6^	2.28 × 10^−3^	0.82	2.16 × 10^6^	1.34 × 10^−2^	6.22
HX001-033	3.10 × 10^6^	1.29 × 10^−3^	0.42	2.83 × 10^6^	4.37 × 10^−2^	15.5
HX001-034	5.10 × 10^6^	3.07 × 10^−3^	0.60	2.64 × 10^6^	1.36 × 10^−2^	5.16

### Structural Investigations of Antibody Neutralization.

**Fig. 3.**
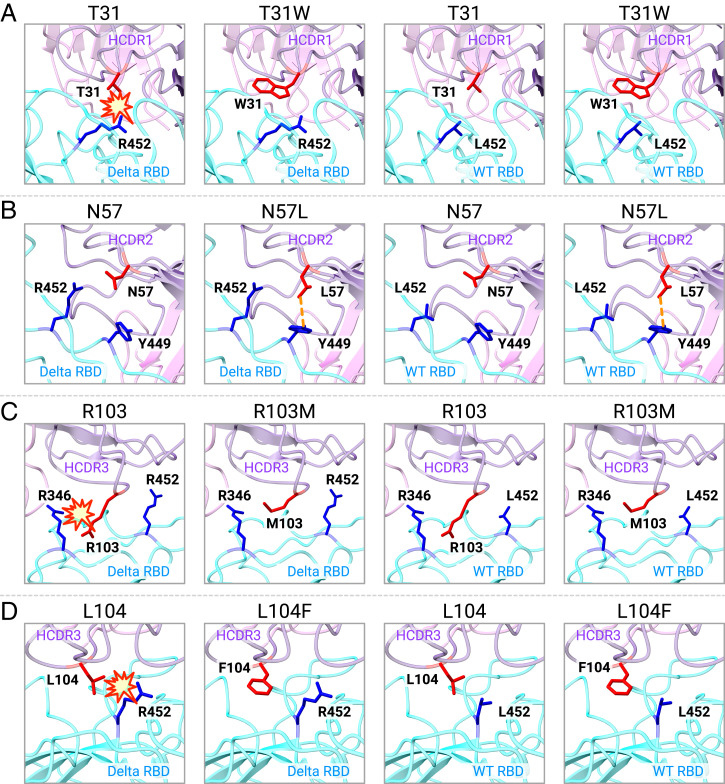
Predicted structure of important mutations on optimized antibodies. (*A*) Predicted structure of interactions between original antibody carrying T31 (red) or optimized antibody carrying W31 (red) with related residue R452/L452 (blue) on Delta/WT RBD (cyan); heavy chain is labeled in purple, and light chain is labeled in pink. (*B*) Predicted structure of interactions between original antibody carrying N57 (red) or optimized antibody carrying L57 (red) with Y449 (blue) and R452/L452 (blue) on Delta/WT RBD. (*C*) Predicted structure of interactions between original antibody carrying R103 (red) or optimized antibody carrying M103 (red) with related residues R346 (blue) and R452/L452 (blue) on Delta/WT RBD. (*D*) Predicted structure of interactions between original antibody carrying L104 (red) or optimized antibody carrying F104 (red) with R452/L452 (blue) on Delta/WT RBD.

To help explain why our predicted mutations could improve the neutralizing activity from a structural perspective, we used Rosetta to predict the complex structures of WT/Delta RBD with antibodies carrying four critical single mutations: T31W, N57L, R103M, or L104F. As shown in Fig. 3*A*, T31 on the original antibody HCDR1 might cause steric clash with the sidechain of R452 on the Delta RBD because the minimal distance between the heavy atoms on two sidechains is within 4 Å. After substituting T31 with W31, which does not have a large sidechain, we no longer see the steric clash with R452 on the Delta RBD. As shown in Fig. 3*B*, N57 on HCDR2 is not on the Delta RBD interaction interface and has no direct contact with Y449 or R452. After substituting N57 with L57, we observed a new interaction between L57’s sidechain and Y449, which potentially explains the enhanced binding affinity of this mutation. As shown in Fig. 3*C*, R103 on the original antibody HCDR3 is spatially very close to the Delta RBD, which may also induce a steric clash with R346. Since both R103 and R346 have very long sidechains and carry positive charges, proximity between the two may introduce strong repulsion which may greatly reduce the binding affinity between the antibody and the antigen. After substituting M103, which has a much smaller sidechain, we no longer observe a direct interaction with R346 on the Delta RBD. This factor might explain the greatly improved neutralization against the Delta variant. Similarly, we can also see that the substitution of L104 for F104 on the HCDR3 might also remove a potential steric clash with R452 on the Delta RBD and thus improve binding and neutralization (Fig. 3*D*).

### Generalization to Omicron Mutations.

A new SARS-CoV-2 variant Omicron emerged in South Africa at the end of November 2021. Omicron carries 36 total mutations in the spike protein and 15 mutations in the RBD, which greatly changed the immunogenicity of the RBD and caused many neutralizing antibodies to lose their neutralization (45–47). Through structure analysis, we found that the G446S mutation was located on the epitope of antibody P36-5D2, and N440K was near (within 5 Å) the epitope. Thus, we applied our deep learning approach to further optimize the antibody sequence HX001-020. The best mutations identified include N92F (HX001-035), G93M (HX001-036), Y94G (HX001-037), and Y74D (HX001-038) in LCDR3. We also constructed a double mutant, HX001-039 (N92F/Y94G), and a triple mutant (N92F/G93M/Y94G). HX001-041 was not produced. It can be seen from Fig. 2*C* that the optimized antibodies were onefold to threefold higher than HX-020 in neutralizing against N440K, and 20- to 100-fold stronger in the pseudotyped virus carrying the G446S mutation. These results demonstrate that adaptive deep learning optimization holds promise for designing potent antibodies against Omicron and future emerging variants.

### Summary.

Collectively, the deep learning platform presented here efficiently optimized the original antibody to obtain broader and more potent neutralizing activity against SARS-CoV-2 variants in only 2 wk for each single-round optimization. Optimized antibodies with single mutations or their combinations can achieve 10- to 600-fold improvement in neutralizing activity against SARS-CoV-2 variants, including Delta. In all optimized antibodies, six antibodies (HX001-013, HX001-015, HX001-020, HX001-024, HX001-033, and HX001-034) had the most potent and broad neutralizing activity, with an IC_50_ between 0.006 μg/mL and 0.017 μg/mL, indicating the capability of our deep learning method to predict mutations with improved neutralizing ability of antibodies against WT or other variants.

## Discussion

In this study, we have reported the efficient optimization of a single original antibody P36-5D2 to obtain broader and more potent neutralizing ability against multiple SARS-CoV-2 variants via a cutting-edge, deep learning guided approach. Based on the crystal structure of the interaction of the RBD and antibodies, and neutralizing data, our geometric neural network model created libraries of CDR mutations and ranked each mutation according to its contribution to binding affinity and structural stability. Through an iterative optimization procedure, we found that optimized antibodies exhibited broader and much more potent neutralizing activity compared to the original antibody. More detailed neutralization assays and binding affinity analyses by SPR further proved that optimized antibodies had hundredfold improvements against specific viral strains.

By deep learning and computational modeling of optimized antibodies of SARS-CoV-2 RBD variants, we observed that predicted optimized antibody candidates could successfully abrogate preexisting steric clashes with RBD at the interface. Some other mutations significantly increase the number of interaction sites, expanding antibody epitopes to more regions on the surface of RBD. Interestingly, a few good mutations were located away from the antibody–RBD interface, which we speculate improve antibody stability and increase CDRs conformational exposure to enhance the neutralization. These results strongly suggested that our model can successfully predict potential modifications of antibodies that are critical to bind to an antigen, with increased structural stability and outstanding neutralizing activity. Surprisingly, peripheral sites not on the binding interface have also been picked up by our deep learning model which seem to be a challenge for conventional structural analysis. Our deep learning model exhibits superior advantages in antibody prediction and optimization for more broader and potent neutralization.

As the virus continues to evolve, more and more diverse variants will emerge in the infected population with greater immune pressure. Many such variants, including Delta and the most recent Omicron, have adopted different mutation strategies to evade immune recognition, leading to the failure of neutralizing antibodies and vaccines (18, 23, 45–48). Undoubtedly, it is urgent to develop and optimize antibodies to overcome emerging or future variants with broad neutralization activities. The conventional in vitro affinity maturation usually takes a long time with poor efficiency due to its small library content, incompatible optimization for multiple goals, and other technical obstacles (32, 49). Directed maturation toward a new variant may also cause the antibody to lose neutralization against previous variants. One important aspect of our approach is that our integrated approach is able to simultaneously optimize binding or neutralization against multiple RBD variants. Based on a deep learning method, we are able to select promising CDR mutation candidates and effectively combine them to achieve efficient antibody optimization. The time spent for each round of the optimization is about 2 wk, most of which is for experimental mutant construction and neutralization assays, much faster than conventional affinity maturation procedures.

Based on our platform, it would even be feasible to train a pool of antibodies that target different potential variants. It is also critical to develop a system to predict virus variants with potential immune escape capabilities such as in Hie et al. (22), even before they emerge. Altogether, we believe that further development of these methods will allow us to predict and keep pace with viral evolution.

## Methods

### Building In Silico Mutation Libraries and Predicted Structures.

Variant-specific amino acid substitutions in the RBD were first created to build complexes with different antigen variants. For each antigen variant, single-point mutations in CDR of the antibody were enumerated. The structures of mutated complexes were sampled using the Rosetta relax and Cartesian ΔΔG programs (43). Point mutations with better predicted binding affinity and experimental neutralization were combined to double and triple mutations, which go through the next round of selection.

### Extraction of Interresidue Interaction Features by Geometric Neural Networks.

Each residue in the complex structure was encoded into a vector representation using a geometric attention network (34) designed to capture interresidue interactions. A residue was represented by its Cα coordinate denoted as $t_{i}$ and an orientation matrix $R_{i}$ defined as

$$\begin{array}{c} v_{i1}=p_{i}^{\left(N\right)}-t_{i},\;v_{i2}=p_{i}^{\left(C\right)}-t_{i},\\ u_{i1},u_{i2}=\left(v_{i1},v_{i2}\right),\\ R_{i}=\left[\frac{u_{i1}}{\left\|u_{i1}\right\|}\;\frac{u_{i2}}{\left\|u_{i2}\right\|}\;\frac{u_{i1}}{\left\|u_{i1}\right\|}\times \frac{u_{i2}}{\left\|u_{i2}\right\|}\;\right], \end{array}$$

where $p_{i}^{\left(N\right)}$ is the position of N, $p_{i}^{\left(C\right)}$ is the position of C, and “GramSchmidt” denotes the Gram–Schmidt process for orthogonalization, where, here, *u_i_*_1_, *u_i_*_2_ are the orthogonal vectors. Each residue was also assigned an initial feature $h_{i}^{\left(0\right)}$ encoding its amino acid type and the local geometry of its atoms,

$$h_{i}^{\left(0\right)}=MLP_{\mathrm{amino}\;\mathrm{acid}\left(i\right)}\left(R_{i}^{T}\left(p_{i}^{\left(a\right)}-t_{i}\right)\;|\;a\in \left\{N,C\alpha ,C,O,C\beta ,\ldots \right\}\right),$$

where each amino acid type was encoded by a separate multilayer perceptron (MLP) with local coordinates of atoms as input. Each residue pair was represented as a vector $z_{ij}$ indicating the relative position of the two residues. The feature vector of a residue was updated iteratively by the multihead attention network (the computational components, or heads, of the transformer network that are repeated multiple times in parallel). The attention weight was formulated by considering residue features, pairwise features, and distances,

$$\begin{array}{c} \beta_{ij}^{h}=\frac{1}{d}L\mathrm{inear}\left(h_{i}^{\left(l\right)}\right)\cdot \mathrm{Linear}\left(h_{j}^{\left(l\right)}\right),\\ \gamma_{ij}^{h}=\mathrm{Linear}\left(z_{ij}\right),\\ \delta_{ij}^{h}=w_{h}\|p_{i}^{\left(C\beta \right)}-p_{j}^{\left(C\beta \right)}\|,\\ \alpha_{ij}^{h}=\mathrm{Softma}x_{j}\left(\beta_{ij}^{h}+\gamma_{ij}^{h}+\delta_{ij}^{h}\right), \end{array}$$

where h denotes the number of the attention head, d is the dimension of $h_{i}^{\left(l\right)}$, and $w_{h}$ is a learnable factor. The message passed from residue j to residue i was formulated as

$$\begin{array}{c} u_{i}^{h}=\underset{j}{\sum }\alpha_{ij}^{h}\;\text{Linear}\left(h_{i}^{l}\right),\\ v_{i}^{h}=\underset{j}{\sum }\alpha_{ij}^{h}\;\text{Linear}\left(z_{ij}^{l}\right),\\ w_{i}^{h}=\underset{j}{\sum }\alpha_{ij}^{h}R_{i}^{T}\left(p_{i}^{\left(C\beta \right)}-p_{j}^{\left(C\beta \right)}\right),\\ m_{i}^{h}=\text{Concat}\left(u_{i}^{h},v_{i}^{h},w_{i}^{h}\right). \end{array}$$

Finally, message vectors $m_{i}^{h}$ from different heads were concatenated to update residue features with a residual connection and a layer normalization step (which directly estimates the normalization statistics from the summed inputs to the neurons within a hidden layer so the normalization does not introduce any new dependencies between training cases). By exchanging information among residues, the final feature vector of each residue $h_{i}^{\left(L\right)}$ encodes its interaction with surrounding residues and will subsequently be used to predict the change in binding affinity.

### Prediction of the Change in Binding Affinity upon Mutation.

Residues in the WT complex and the mutant complex were encoded using the aforementioned geometric neural network. As a complement to the features output by the network, physical energy values of a complex calculated by Rosetta were also included as a feature shared across residues in the complex. Let $h_{i}^{wt}$ denote the feature of the i th residue in the WT complex, and let $h_{i}^{\mathrm{mut}}$ denote the feature of its counterpart in the mutant complex. These features were used as input to the following antisymmetric network so as to predict the difference in binding affinity between the two complexes:

$$\begin{array}{c} x_{i}={\text{MLP}}_{1}\left(h_{i}^{\text{wt}},h_{i}^{\text{mut}}\right)-{\text{MLP}}_{1}\left(h_{i}^{\text{mut}},h_{i}^{\text{wt}}\right),\\ D=W\underset{i}{\sum }x_{i}, \end{array}$$

where, $MLP_{1}$ is a standard MLP network, and W is a trainable weight matrix.

### Model Training.

The training and validation datasets were constructed from the SKEMPI V2.0 dataset (38), the largest antibody–antigen binding affinity dataset, which contains 342 complexes and 5,318 mutations in total. Mutant structures were sampled by the Rosetta Cartesian ΔΔG program. The dataset was split evenly into five subsets without overlapping complexes. Accordingly, five models were trained, using each subset for validation. For each chain in a complex, we chose only the 48 residues spatially closest to the other chain to train the model, because it was sufficient to consider only residues on or close to the interface. The loss function we used for training was the mean-squared error (MSE) between the predicted ΔΔG values and the experimentally measured values. The model was trained for 100,000 iterations using the Adam optimizer at a learning rate of 1e-4 until convergence. Validation was performed every thousand training steps to monitor the optimization procedure. The learning rate was reduced by half if the lowest validation loss did not drop for 10 validation iterations.

### Evaluating Model Performance.

The model was evaluated by split-by-complex fivefold cross-validation over the entire SKEMPI V2.0 dataset. A subset consisting of 1,131 single-point mutations (S1131) (39) was used to benchmark the model and other baselines (50, 51). An ensemble model that averages the prediction of our model, GeoPPI, and Rosetta was also evaluated. Pearson correlation between the predicted ΔΔG values by different methods and real ΔΔG is reported in *SI Appendix*, Table S1. Figure plots of ΔΔG predicted by the model and experimental ΔΔG are reported in Fig. 1*B*. An additional subset consisting of multipoint mutations (M1707) (40) was also used as a benchmark; Pearson correlation on this subset is reported in *SI Appendix*, Table S1. Overall, our model's predictions were highly correlated with experimental results.

### Ranking Mutations by Model Ensemble.

Mutants in CDRs were scored using the average predicted ΔΔG from an ensemble of five trained models, as the models were trained in a fivefold cross-validation manner. Mutations were grouped by antigen variants and sorted in ascending order; those with lower ΔΔG values were more favorable. We also used GeoPPI as another ΔΔG predictor for ranking mutations in PPI complexes based on a pretrained graph neural network and a random forest regressor (41). The ΔΔG values reported by the Rosetta Cartesian ΔΔG program were also employed to rank mutations. The average of three rankings serves as the criteria for selecting candidate mutations.

The initial single-point mutation library was built as follows: 1) All single-point mutations in HCDRs were enumerated and scored; 2) all double-point mutations from the top single-point mutations were enumerated; and 3) all single and double mutations were ranked, and the top mutations on three CDR-H loops were selected as the initial mutations, including T30P, T31W, Y32O, N52D, N53F, N55L, N57L, G100P, R103M, L104F, and Q105F.

### Cell Lines.

HEK293T cells (ATCC), HeLa cells expressing hACE2 orthologs (kindly provided by Qiang Ding, Center for Infectious Disease Research, School of Medicine, Tsinghua University, Beijing, China) were maintained at 37 °C in 5% CO_2_ in Dulbecco’s minimal essential medium containing 10% (vol/vol) heat-inactivated fetal bovine serum and 100 U/mL of penicillin–streptomycin. FreeStyle 293F cells (Thermo Fisher Scientific, R79007) were maintained at 37 °C in 5% CO_2_.

### Antibody and Fab Production.

Antibodies and Fab production were conducted as previously described (33, 52). Reference REGN10987 were synthesized according to the sequences released in the Protein Data Bank (44, 53–56). Antibodies were produced by transient transfection of HEK 293F cells (Life Technologies) using plasmids expressing the heavy and light chain by 1 mg/mL polyetherimide (Sigma). After 5 d, the supernatant was collected and purified with protein A. Antibodies were eluted from protein A with elution buffer (0.3 M glycine, pH 2.0), followed by dialyzing into phosphate-buffered saline (PBS).

### Production of Pseudotyped SARS-CoV-2.

The WT pseudotyped virus used throughout the analysis was the prototype strain (GenBank: MN908947.3) with a D614G mutation (WT D614G). Variants were extracted from the Global Initiative on Sharing All Influenza Data (GISAID) website. The Alpha variant (GISAID: EPI_ISL_601443) was constructed with a total of nine mutations (69-70del, 144del, N501Y, A570D, D614G, P681H, T716I, S982A, and D1118H). The Beta variant (GISAID: EPI_ISL_700450) was constructed with 10 mutations: L18F, D80A, D215G, 242-244del, S305T, K417N, E484K, N501Y, D614G, and A701V. The Gamma variant (GISAID: EPI_ISL_792681) was constructed with 12 mutations: L18F, T20N, P26S, D138Y, R190S, K417T, E484K, N501Y, D614G, H655Y, T1027I, and V1176F. The Delta variant (GISAID: EPI_ISL_1534938) was constructed with 10 mutations: T19R, G142D, 156-157del, R158G, A222V, L452R, T478K, D614G, P681R, and D950N. The variant Delta Plus (GISAID: EPI_ISL_3019629) was constructed with 11 mutations: 19R, G142D, 156-157del, R158G, A222V, K417N, L452R, T478K, D614G, P681R, and D950N. The Kappa variant (GISAID: EPI_ISL_1384866) was constructed with eight mutations: T95I, G142D, E154L, L452R, E484Q, D614G, P681R, and N1071H. The Eta variant (GISAID: EPI_ISL_2885901) was constructed with eight mutations: Q52R, A67V, 69-70del, 144del, E484K, D614G, Q677H, and F888L. The Epsilon variant (GISAID: EPI_ISL_2922315) was constructed with four mutations: S13I, W152C, L452R, and D614G. The single mutation N439K was introduced into the pcDNA3.1 vector encoding WT D614G using QuikChange site-directed mutagenesis (Agilent 210519). The gene of variants were synthesized in Genewiz, Inc. WT, and mutated SARS-CoV-2 pseudotyped viruses were generated by cotransfection of human immunodeficiency virus backbones expressing fireﬂy luciferase (pNL43R-E-luciferase) and pcDNA3.1 (Invitrogen) expression vectors encoding the respective spike proteins into 293T cells (ATCC) (20). Viral supernatants were collected 48 h later. Viral titers were measured as the luciferase activity in relative light units (Bright-Glo Luciferase Assay Vector System; Promega Biosciences).

### Neutralization Activity of Monoclonal Antibodies against Pseudotyped SARS-CoV-2.

Neutralization assays were conducted as previously described (33). Serial dilutions of monoclonal antibodies (mAbs) were prepared with the highest concentration of 5 or 50 μg/mL. WT or mutated spike pseudotyped viruses were incubated with mAbs at 37 °C for 1 h. HeLa-hACE2 cells (1.5 × 10^4^ per well) were then added into the mixture and incubated at 37 °C for 48 h to 60 h. Then the luciferase activity was measured after cell lysis. The percent of neutralization was determined by comparing with the virus control.

### Antibody Binding Kinetics Measured by SPR.

The binding kinetics of mAbs to SARS-CoV-2 WT or Delta RBD monomer were analyzed using SPR (Biacore 8K; GE Healthcare). Specifically, recombinant protein A (Sino Biological) was immobilized to a CM5 sensor chip. The mAbs (2 μg/mL) were captured by recombinant protein A, and then serial dilutions of SARS-CoV-2 WT/Delta RBD with highest concentration of 200 nM were run at a flow rate of 30 μL/min in PBST buffer (1×PBS and 0.05% [vol/vol] Tween-20). The resulting data were fitted to a 1:1 binding model using the Biacore 8K Evaluation software (GE Healthcare).

### Quantification and Statistical Analysis.

The technical and independent experiment replicates are indicated in the figure legends. IC_50_ of mAbs were calculated by the four-parameter dose inhibition equation in using Graphpad Prism 9.0.

## Supplementary Material

Supplementary File

## Data Availability

All study data are included in the article and/or *SI Appendix*. The code can be found in GitHub at https://github.com/HeliXonProtein/binding-ddg-predictor.
