# Supplementary material for: Deep learning guided optimization of human antibody against SARS-CoV-2 variants with broad neutralization
Source: Proc Natl Acad Sci U S A. 2022 Mar 1;119(11):e2122954119. doi: 10.1073/pnas.2122954119 (PMC8931377; doi:10.1073/pnas.2122954119)
Supplement: Supplementary File [file pnas.2122954119.sapp.pdf]

Supplementary Information for

Deep learning guided optimization of human antibody against SARS-CoV-2 variants with broad neutralization

Sisi Shan, Shitong Luo, Ziqing Yang, Junxian Hong, Yufeng Su, Fan Ding, Lili Fu, Chenyu Li, Peng Chen, Jianzhu Ma, Xuanling Shi, Qi Zhang, Bonnie Berger, Linqi Zhang, Jian Peng

Bonnie Berger, Linqi Zhang, Jian Peng
Email: bab@mit.edu, zhanglinqi@tsinghua.edu.cn, or jianpeng@helixon.com

This PDF file includes:

Figures S1 to S3
Table S1

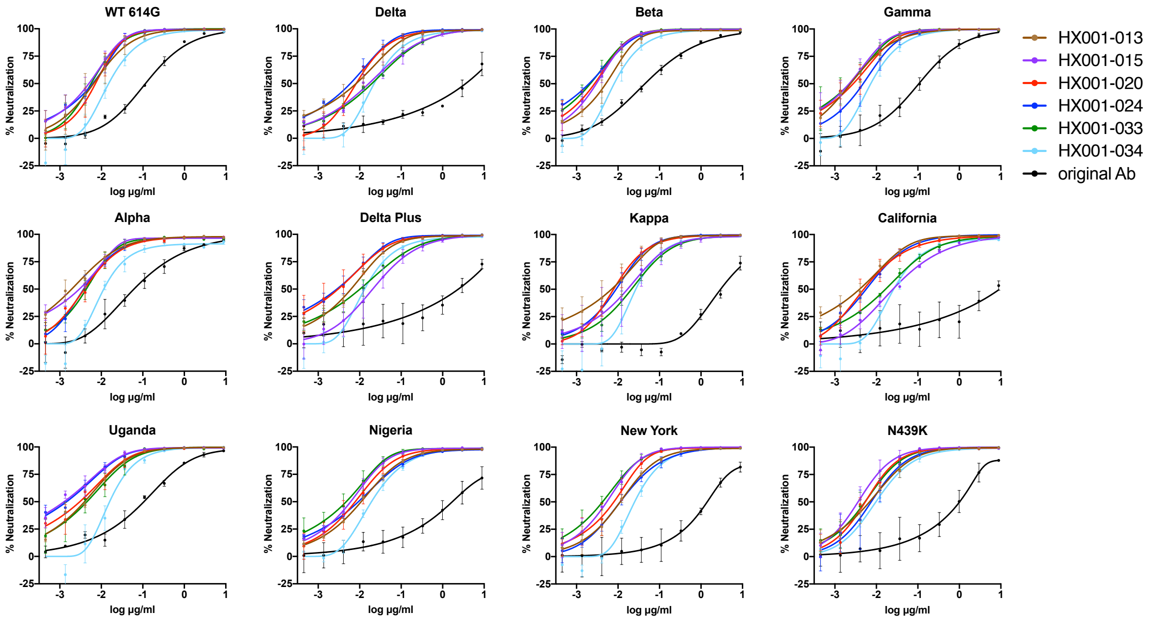

Figure S1. IC50 values of SARS-CoV-2 neutralizing antibodies against pseudotyped SARS-CoV-2 variants. Mutations of each variant on RBD are indicated.

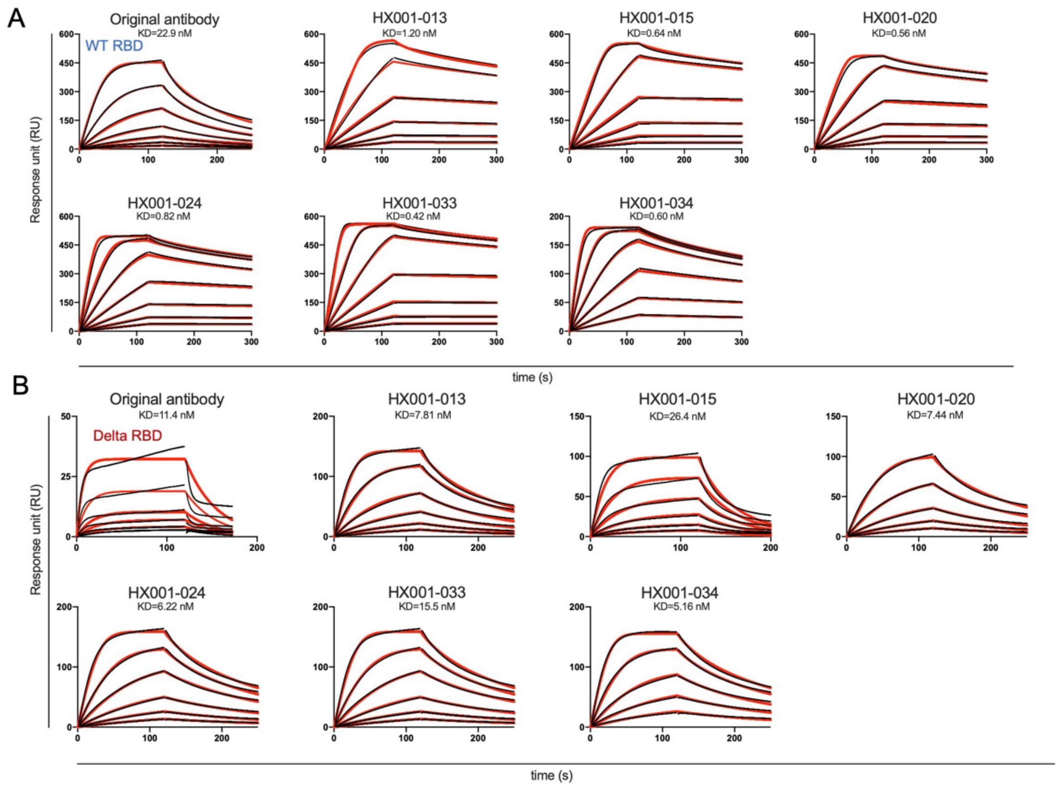

Figure S2. Binding kinetics of original antibody and optimized antibodies with SARS-CoV-2 RBD measured by SPR. The black lines indicate the experimentally derived curves while the red lines represent fitted curves based on the experimental data.

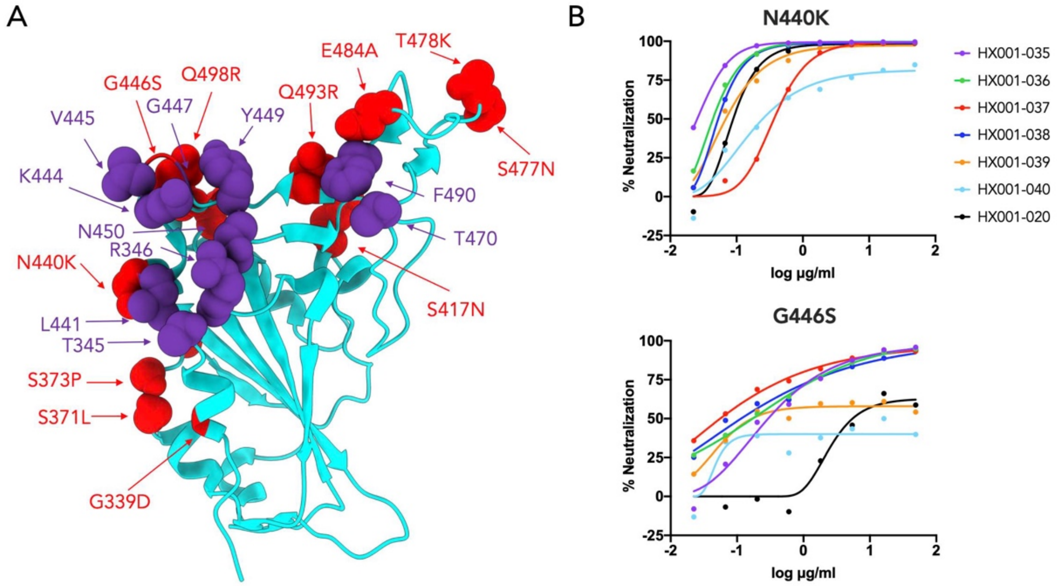

Figure S3. Optimization of antibody HX001-020 against Omicron mutations. (A) The cartoon structure shows the epitope of P36-5D2 (purple) on RBD (cyan), and the mutations of omicron in RBD (red). G446 is located on the epitope and G446S was mutated in omicron. (2) Neutralization curves of original antibody HX001-020 and optimized antibodies HX001-035, HX001-036, HX001-037, HX001-038, HX001-039, HX001-035 against pseudotyped SARS-CoV-2 carrying N440K or G446S.

Table S1. Model Performance on SKEMPI 2.0 Dataset.

Method	S1131	M1707
Ours	0.65	0.59
GeoPPI [37]	0.58	0.74
TopGBT [46]	0.32	-
TopNetTree [46]	0.29	-
Rosetta	0.36	-
FoldX [47]	0.46	0.49
Ensemble	0.68	-

Model performance on SKEMPI 2.0 dataset. Pearson correlations between model-predicted ddG and experimental ddG are reported.
